# Exogenous abscisic acid inhibits leaf elevation during shade avoidance response in *Arabidopsis thaliana*

**DOI:** 10.17912/micropub.biology.001972

**Published:** 2026-02-03

**Authors:** Brett E. Harris, Helena E. Heiberger, ByungHoon B. Kim

**Affiliations:** 1 Department of Biology, University of North Alabama, Florence, Alabama, United States

## Abstract

Plant shade avoidance response includes elongated hypocotyls and petioles as well as increased leaf elevation angles. Our time-course image analyses indicate that the phytohormone abscisic acid (ABA) affects leaf elevation angles in Arabidopsis. While mutants with impaired ABA production ( *aba1-6* ) or with insensitivity to ABA ( *abi5-10* ) did not fully increase the angles under shade avoidance conditions, a higher concentration of exogenous ABA inhibited leaf elevation under the same conditions. These suggest that certain levels of ABA production and sensing are required for leaf elevation during shade avoidance response, whereas higher concentrations of ABA can inhibit the process.

**Figure 1. Time-course measurements of leaf elevation indexes in wild-type plants, aba1-6 and abi5-10 mutants&nbsp;:**
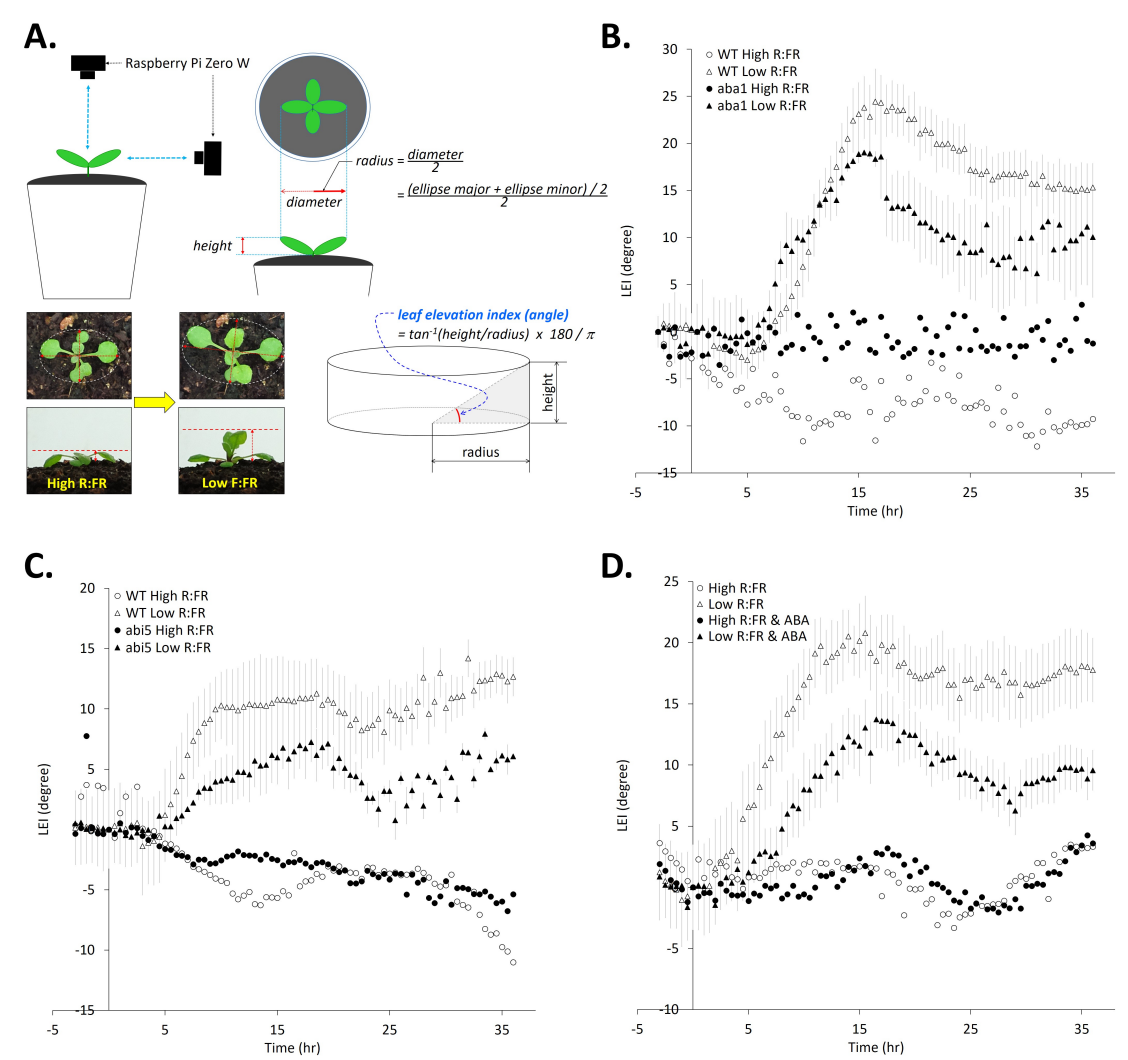
(A) The concept of leaf elevation index. (B) Leaf elevation response in *aba1-6* mutants during shade avoidance response (WT High R:FR, n=3; WT Low R:FR, n=6; *abi1-6* High R:FR, n=2; *abi1-6* low R:FR, n=3). (C) Leaf elevation response in *abi5-10* mutants during shade avoidance response (WT High R:FR, n=3; WT Low R:FR, n=3; *abi5-10* High R:FR, n=3; *abi5-10* low R:FR, n=3). (D) The effect of ABA on leaf elevation during shade avoidance response in wild-type plants (High R:FR, n=6; Low R:FR, n=9; High R:FR & ABA, n=4; low R:FR & ABA, n=8). ABA (100 μM) was sprayed every 24 hr. All plants were treated with indicated conditions at 0 hr time point. Error bars indicate standard error. To avoid visual complexity, the error bars for the data from high R:FR-treated plants were not shown.

## Description

As a source of energy, light has a significant impact on plant development. When plants are grown in dense vegetation, they increase the length of hypocotyls and petioles as well as the leaf elevation angles to compete with other plants for sunlight. Such a set of responses is collectively called shade avoidance response (Casal and Fankhauser, 2023). Photoreceptors can sense fluctuations in light quality, the ratio of red light (R; λ = 660 nm) to far-red light (FR; λ = 730 nm). A lower ratio (R:FR) is created in an area of dense vegetation due to the selective absorption of red light by chlorophylls in other plants, whereas a higher R:FR ratio is created in the area where plant density is low. Phytochromes exist in two photo-convertible forms, including an inactive red-light absorbing form (P _r_ ) and an active far-red light absorbing form (P _fr_ ). Under high R:FR conditions, more P _fr_ forms exist in plants that lead to normal photomorphogenic development, while more P _r_ forms exist under low R:FR conditions which elicit shade avoidance response. Particularly, an elevated leaf angle is achieved through enhanced production of auxin in the leaf, which is transported to the abaxial side of the petiole, which in turn elicits unequal elongation of cells at the base of the petiole (Gao et al., 2020). On the other hand, the stress hormone abscisic acid (ABA) has also been known to affect elongational growth and leaf angles during the plant growth under shade (Mullen et al., 2006; Benschop et al., 2007; Michaud et. al, 2023; van Zanten et al, 2009).&nbsp;&nbsp;Especially, Michaud et al. (2023) showed that ABA deficient mutants *aba2* ( At1g52340 ) and *nced3nced5* ( AT3G14440 ; AT1G30100 ) exhibited reduction of leaf elevation response under low R:FR conditions, suggesting that ABA is necessary for the full range of leaf elevation response.

In the present study, we investigated the leaf elevation dynamics in different ABA mutants and wild-type plants under low R:FR conditions. To this end, a simple concept of leaf elevation index (LEI) was introduced (Kim, 2025) and combined with time-course image analyses through a computational approach described before (Kim, 2025). A *leaf elevation index* of an individual plant was computed by using the plant's height and the radius measured by analyzing each image during the time-course ( Fig. 1A ). Previously, this approach was successfully used to detect the differences in dynamics of leaf elevation levels of wild-type plants (Col-0) and *pif* mutants ( *pif4-2pif5-3* and *pif7-2;* At2g43010 ; At3g59060 ; At5g61270 ) during shade avoidance response (Kim, 2025).

First, we investigated the leaf elevation dynamics in ABA-deficient ( *aba1-6;* AT5G67030 ) and ABA-insensitive ( *abi5-10* ; AT2G36270 ) mutants under low R:FR conditions. Both *aba1-6* mutants and *abi5-10* mutants exhibited lower levels of leaf elevation response when compared with wild-type plants (Col-0; Fig. 1B and 1C). Furthermore, the response kinetics of *abi5-10* mutants indicate slower responses in leaf elevation process than in the wild-type plants ( Fig. 1C ), suggesting that ABA is required for plants to fully elevate the leaves in response to low R:FR conditions. On the other hand, when exogenous ABA (100 μM) was sprayed to wild-type plants, the levels of leaf elevation under low R:FR conditions were not as pronounced as in the wild-type plants without an ABA treatment ( Fig. 1D ). Moreover, the ABA-treated samples responded slower than the non-ABA-treated samples (lower slope; peak at 17 hr vs. 11.5 hr; Fig. 1D ). This suggests that ABA inhibits the leaf elevation process under low R:FR conditions.

Our results suggest that proper production and sensing of endogenous ABA hormone is required for normal leaf elevation during shade avoidance response ( Fig. 1B and 1C) while exogenous ABA inhibits this process ( Fig. 1D ). Although these results seem to be contradictory to each other, ABA has been recognized as both an inhibitor and promoter of growth under different conditions (reviewed in Brookbank et al., 2021). More recently, similar measurements of leaf elevation angles in ABA deficient mutants *aba2* and *nced3nced5* also indicated reduction of leaf elevation response under low R:FR conditions (Michaud et al., 2023), confirming our results from *aba1-6* and *abi5-10* mutants studies. In addition, low R:FR conditions elicit ABA production in Arabidopsis plants, which seems to be necessary for highest levels of leaf elevation in wild-type plants (Michaud et al., 2023). However, their experiments with exogenous ABA elicited either no significant effect (1 μM ABA) or slightly delayed response (10 μM ABA) during the leaf elevation under low R:FR conditions (Michaud et al., 2023). The obvious inhibition of leaf elevation in our experiments occurred when the ABA concentration was 100 μM ( Fig. 1D ). Although the exact levels of endogenous ABA contents are elusive, especially when applied exogenously, it appears that the role of ABA in the leaf elevation process during shade avoidance response depends on its concentrations. Lower concentrations may enhance while higher concentrations may inhibit leaf elevation.

ABA production under abiotic stresses might be much higher than the levels induced by low R:FR. Such high levels of ABA induced by environmental stresses can inhibit elongational growth or even lead to a growth arrest ( Fig. 1D ; Chapin, 1991). Although the conditions were not the same, it has been reported that the levels of endogenous ABA contents after 1 hour of dehydration treatment were dramatically higher than those with dark treatment (Kim and von Arnim, 2006). Taken together, the effect of ABA on leaf elevation under low R:FR conditions seems to be dosage dependent. Since the endogenous levels of ABA are influenced by the environmental contexts around the plant, the levels of leaf elevation under low R:FR conditions may also depend on the environmental contexts.&nbsp;

## Methods

**Plant material and growth** &nbsp;

Wild-type (WT) and mutant ( *aba1-6* , *abi5-10* ) plants used in this experiment were *Arabidopsis thaliana* Columbia-0 (Col-0). Seeds were surface sterilized with a 30% Clorox® solution with 0.1% Triton X-100 and rinsed with autoclaved water. Then, the seeds were sown on Murashige-Skoog medium with 0.8% agar. They were kept in the fridge for a week for stratification for even germination. Then, they were grown at 23°C under normal light conditions (80 µmol/m ^2^ /s; R:FR = 3) for a week before transplanted onto soil and grown for another week under the same conditions. The pots were placed in a growth chamber (continuous light; 120 µmol/m ^2^ /s; R:FR = 2.5) installed with Raspberry Pi units (Kim, 2005). After 24 hr of acclimation period for the slight environmental change, far-red LED lights were supplemented for low R:FR treatments (R:FR ratio = 0.36), while plants for high R:FR conditions were kept without the supplemental LED lights (R:FR ratio = 2.5). ABA treatment was done by spraying 100 µM ABA solution using a small perfume sprayer every 24 hours. The soil is kept moisturized by placing the pot on a tall Petri dish filled with water.&nbsp;&nbsp;

**Image Analysis** &nbsp;

Time-lapse images were taken every 30 minutes by using *Raspberry Pi Zero W* microcomputers as described earlier (Kim, 2025). For every plant at a specific time point, a top view and a side view pictures were taken. The software *PlantCV* was used to determine the plants' height, ellipse major, and ellipse minor (Berry et al., 2018), which were converted to millimeters using NIH ImageJ (Schneider et al., 2012). Further analyses were carried out using Microsoft Excel®. The mean value of the ellipse major and the ellipse minor was used as the diameter of the rosette, and the radius was determined from this diameter. The leaf elevation index (LEI) was determined from the radius (r) and height (h) of the hypothetical cylinder (LEI = tan ^-1^ (h/r) x 180/π) ( Fig. 1A ). Even though this approach does not measure the actual elevation angle of a particular leaf, it assigns a single representative value (LEI) for a plant. This was useful in estimating the leaf elevation status of a whole plant (Kim, 2025). Mean values and standard errors were calculated. In each data set for a particular treatment, the average LEI values were normalized so that the LEI at the beginning of the treatment is set to zero (LEI=0 at 0 min of treatment).&nbsp;

## Reagents

*Arabidopsis thaliana* wild type and mutant plants used in this study&nbsp;

**Table:** 

Ecotype&nbsp;	Genotype&nbsp;	Available From&nbsp;
Columbia&nbsp;	Wild-type&nbsp;(Col-0)	&nbsp;
Columbia&nbsp;	*aba1-6* &nbsp;	ABRC # CS3772&nbsp;
Columbia&nbsp;	*abi5-10* &nbsp;	ABRC # SALK_200891C&nbsp;

&nbsp;
